# New fluoroscopic imaging technique for investigation of 6DOF knee kinematics during treadmill gait

**DOI:** 10.1186/1749-799X-4-6

**Published:** 2009-03-13

**Authors:** Guoan Li, Michal Kozanek, Ali Hosseini, Fang Liu, Samuel K Van de Velde, Harry E Rubash

**Affiliations:** 1 Bioengineering Laboratory, GRJ 1215, Massachusetts General Hospital, 55 Fruit Street, Boston, MA 02114, USA

## Abstract

**Introduction:**

This report presents a new imaging technique for non-invasive study of six degrees of freedom (DOF) knee kinematics during treadmill gait.

**Materials and methods:**

A treadmill was integrated into a dual fluoroscopic imaging system (DFIS) to formulate a gait analysis system. To demonstrate the application of the system, a healthy subject walked on the treadmill at four different speeds (1.5, 2.0, 2.5 and 3.0 MPH) while the DFIS captured the knee motion during three strides under each speed. Characters of knee joint motion were analyzed in 6DOF during the treadmill walking.

**Results:**

The speed of the knee motion was lower than that of the treadmill. Flexion amplitudes increased with increasing walking speed. Motion patterns in other DOF were not affected by increase in walking speed. The motion character was repeatable under each treadmill speed.

**Conclusion:**

The presented technique can be used to accurately measure the 6DOF knee kinematics at normal walking speeds.

## Introduction

Accurate data of six degrees-of-freedom (6DOF) knee kinematics is instrumental for investigation of biomechanical mechanisms of knee pathology such as osteoarthritis, ligamentous injuries and total knee arthroplasty. Traditional gait analysis used multiple video cameras to track the three-dimensional (3D) motions of reflective markers fixed to the skin[1], which was limited to reveal relative motion of the femoral and tibial bones. Invasive methods, such as using reflective markers directly fixed to bone using a thin rod[2] or opaque markers embedded within the bones, [3-7] were applied to detect bony motion in order to eliminate the effect of skin motion and enhance the accuracy of motion data. In another way, a point-cluster technique, which is noninvasive, has also been proposed to improve the traditional gait analysis method in order to reduce the effect of relative motion of the skin and bones[8].

Recently, fluoroscopic imaging technique, due to its relative accessibility, easiness to operate, and low radiation dosage compared to traditional X-rays, has been used for the analysis of knee joint motion during gait [9-11]. However, the use of just a single image might not detect knee joint motion in the out-of-plane degrees-of-freedom in the same accuracy as compared to the accuracy in in-plane motion[12,13]. In our laboratory, we validated the method using the cine function of two fluoroscopes to simultaneously capture dynamic knee joint motion[14]. This study presents how to use this technique to determine 6DOF knee joint motion during treadmill gait with different speeds.

## Methods

### DFIS Setup

**Figure 1:**
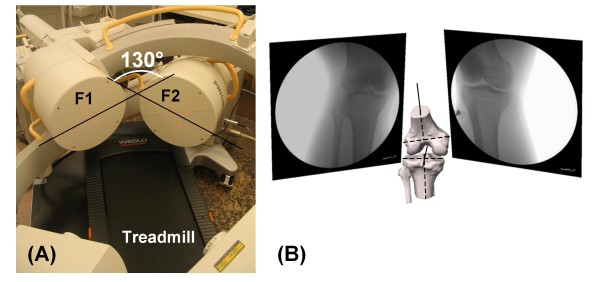
**(A) Setup of the DFIS system and a treadmill**. **(B)** Knee model and virtual DFIS environment for reproduction of in-vivo knee kinematics. A typical knee model is shown after reproducing its position in the virtual environment of the modeling software.

The dual fluoroscopic imaging system (DFIS) setup that was validated previously is used for the treadmill gait analysis (Fig. 1) [14,15]. The DFIS consists of two pulse fluoroscopes (BV Pulsera, Philips) that are set to generate 8 ms width X-ray pulses with an effective dose of 13 mrem per scanning. In this study, the fluoroscopes took 30 evenly distributed snapshot images per second during dynamic knee joint motion.

The diameter of the image intensifier of the fluoroscopes is ~310 mm. In general, given the size of the image intensifier of the fluoroscopes might be difficult to capture the entire range of knee motion during the treadmill gait. Therefore, the two fluoroscopes are positioned so that their intensifiers form an angle between 120 and 130° (Fig. 1). In this setup, the dual fluoroscopic system has a common field of view with a length of ~450 mm. Therefore, the entire knee motion could be captured by both fluoroscopes during the gait cycle.

### Treadmill gait

To demonstrate the methodology of treadmill gait analysis, one healthy subject (male, 45 years old) performed gait on the treadmill at different speeds: 1.5, 2.0, 2.5 and 3.0 mile/hour MPH (or 0.67, 0.89, 1.12 and 1.34 m/s, respectively). Two laser-positioning devices were attached to the two fluoroscopes to help the subject align the target knee (left) within the field of view of the fluoroscopes during gait with the assistance of a technician. The knee was then imaged from heel strike to toe-off during three consecutive strides after about 30 seconds of practice. The subject took 5 minute rest after testing for each speed.

### Reproduction of in-vivo knee kinematics

The anatomic model of the target knee, including the bony geometry of the tibia and femur, was reconstructed by tracing the bony contours on sagittal plane magnetic resonance (MR) images of the knee in solid modeling software (Rhinoceros^®^, Robert McNeal & Associates, Seattle, WA). The MR images were obtained using a 3.0 Tesla MR scanner (MAGNETOM^® ^Trio, Siemens, Erlangen, Germany) while the subject was lying supine with the knee in a relaxed, extended position. The MR scanner employed a 3D double echo water excitation sequence and the following parameters: field-of-view = 160 × 160 × 120 mm, voxel resolution = 0.31 × 0.31 × 1.00 mm, time of repetition (TR) = 24 ms, time of echo (TE) = 6.5 ms, and flip angle = 25°. A joint coordinate system described previously (Fig. 1B) was adopted to determine the 6DOF knee joint kinematics [16].

The model and the dual fluoroscopic images were placed into a virtual DFIS environment where the in-vivo positions of knee were reproduced by matching projections of the models to their outlines on the fluoroscopic images [12]. The knee positions during three strides at each treadmill speed were reproduced. For each stride, the knee position was analyzed at each 10% of the stance phase from heel strike to toe-off.

The average speed of the knee during stance phase was calculated by dividing the maximal traveling distance by the corresponding traveling time. The data on 6DOF knee kinematics, including knee flexion, internal-external tibial rotation, as well as medial-lateral translation and varus-valgus rotation, were analyzed. The repeatability of the treadmill gait was determined by the standard deviation of the three strides of each treadmill speed.

## Results

**Figure 2:**
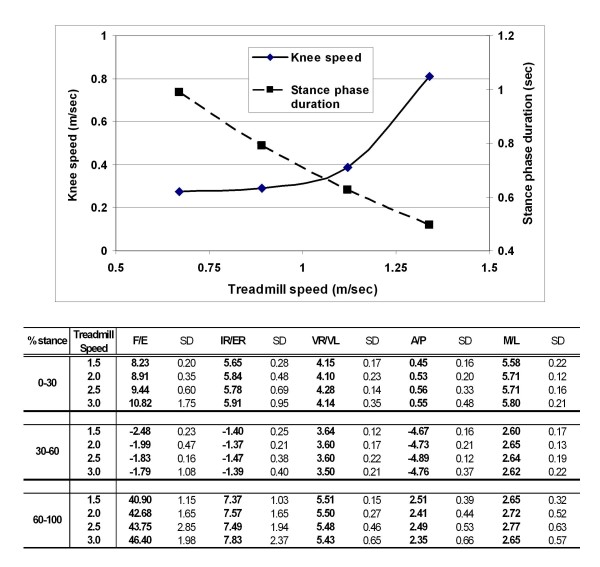
**Top: Average speed of the knee and duration of the stance phase of gait on treadmill at four different walking speeds**. **Bottom**: Peak kinematic values of the knee during different intervals of the stance phase. F/E: flexion (+)/extension(-); IR/ER: internal(-)/external(+) femoral rotation; A/P: anterior(+)/posterior(-) femoral translation; ML: medial(-)/lateral(+) femoral translation; SD: standard deviation.

The duration of the stance phase decreased with the treadmill speed (Fig. 2). At 1.5 MPH, the stance phase time was 0.99 second, while at treadmill speed of 3.0 MPH, the stance phase time decreased to 0.49 second. The average speed of the knee during stance phase was lower than the treadmill speed (Fig. 2). At 1.5 MPH treadmill speed, the knee speed was 0.28 ± 0.02 m/second, only 41% of the treadmill speed. At 2.5 MPH treadmill speed, the knee speed was 0.39 ± 0.05 m/second that was 35% of the treadmill speed. At 3.0 MPH treadmill speed, the knee speed was 0.81 ± 0.02 m/second that was 60% of the treadmill speed.

**Figure 3:**
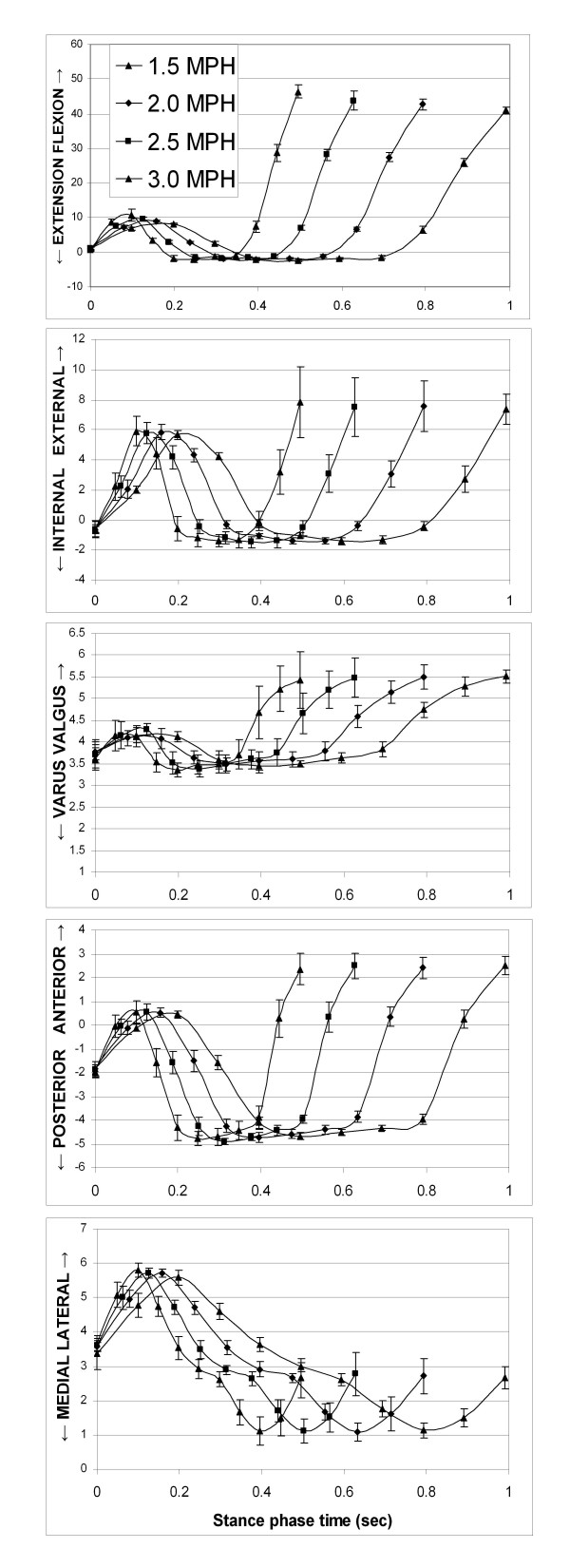
**In-vivo knee kinematics during the stance phase of gait on the treadmill**. The data for translations and internal-external rotation represent motion of the femur with respect to the tibia. The kinematic values are charted as function of time [sec].

Knee kinematics under different treadmill speeds showed similar patterns in both, the rotations and the translations (Fig. 3). After heel strike, the tibia showed an increase in flexion angle to 6.71 ± 0.86° and 13.81 ± 2.73° and an increase in internal tibial rotation to 4.56 ± 0.29° and 5.45 ± 0.76° for the treadmill speeds of 1.5 MPH and 3.0 MPH, respectively (Fig. 2B). During mid-stance, the knee showed maximal hyperextension of about -2.5° and external tibial rotation of about -1.5° at all speeds. At toe-off, the knee had flexion angles of 43.05 ± 2.18° and 52.35 ± 5.09° and internal tibial rotation of 4.73 ± 0.35° and 7.56 ± 1.50° for treadmill speeds of 1.5 and 3.0 MPH, respectively. The knee also showed an increase in valgus rotation after heel strike, a decrease in valgus rotation during stance phase and sharper increase in valgus rotation at toe-off (Fig. 2B and Fig. 3C).

Femoral translations during gait at different speeds showed similar patterns (Figs. 3D and 3E). The femur translated anteriorly during loading response and early midstance and moved posteriorly thereafter until terminal stance when it shifted anteriorly again. In medial-lateral direction, the femur moved laterally during early stance and medially towards toe-off.

## Discussion

This paper introduced the technique of using the DFIS for measurement of 6DOF in-vivo knee kinematics during treadmill gait. The data showed that this technique is feasible to analyze the dynamic knee motion during a wide range of treadmill speeds (up to 3 MPH). Since this technique reproduced the knee positions using 3D anatomic models of the knee, 6DOF tibiofemoral joint kinematics during gait can be obtained.

Few studies have utilized fluoroscopes to investigate human knee motion during gait [17]. For example, Zihlmann et al. [17] moved a fluoroscope to follow the knee motion to overcome the limited field of view of the image intensifier. They estimated an accuracy of 0.2 mm for in-plane translation and of 3.25 mm for out-plane translation and an accuracy of 1.57° for rotation in a knee after total joint arthroplasty during level walking. To overcome the limitation of the image intensifier size in the DFIS set up, the two fluoroscopes are positioned so that their common image zone covers the knee motion during the complete gait cycle on a treadmill [14].

The DFIS has been recently validated to measure dynamic knee motion [14]. Using standard geometry, the sphere positions could be determined with a SD below 0.2 mm when sphere moved at a speed up to 0.5 m/second. The dynamic validation using cadaveric knees, demonstrated that the DFIS on average has an accuracy of less than 0.15 mm and 0.1 mm/s in determining translation and velocity, respectively. Varadarajan et al. [15] demonstrated that the DFIS can measure translation in knee after total joint arthroplasty with an accuracy of less than 0.4 mm at a speed of 0.5 m/s. The accuracy of the DFIS depends on the speed of the moving joint.

The data of this paper revealed that the knee traveling speed is lower than the treadmill speed (Fig. 2). At the treadmill speed of 2.5 MPH, the knee speed during stance phase is less than 0.4 m/second, while at treadmill speed of 3.0 MPH, the knee speed is about 0.8 m/second. Our data also showed that with increasing speed, the amplitude of knee flexion during stance phase increases. This finding is in agreement with other studies in the literature [18-21]. Treadmill gait was also shown to be repeatable across the multiple strides as indicated by the standard deviation calculated from three strides at each treadmill speed.

The pulse imaging character of the fluoroscopes is an important factor for analyzing treadmill gait. In a pulsed fluoroscopic system such as the one used in our set up, the pulse width and frame rate are decoupled parameters. The inverse of frame rate corresponds to time difference between two consecutive images, whereas pulse width corresponds to excitation time for each image. If the rate at which pulses are generated is matched to the rate of acquisition then each image corresponds to a pulse. In this case, pulse width limits the image quality for a given frame rate. Theoretically, the maximal pulse rate (and matched frame rate) is limited by the pulse width. Therefore, a pulse width of 8 ms has a maximum frame rate of 125 frames/second, which is higher than the recommended minimal frame rate of 60 frames/second for gait analysis. However, a reduced rate of image capture (e.g. 15 or 30 pulses/second and matched frame rates) can be employed to limit unnecessary radiation exposure and data processing without adversely affecting image quality. This is because the image quality is actually related to pulse width even though fewer images are taken. Therefore, we could chose to use 15 or 30 pulses/second in our application, depending on the moving speed of the joint.

In summary, this paper introduced the DFIS technique for measurement of 6DOF in-vivo knee kinematics during treadmill gait. The technique showed feasibility to analyze the dynamic knee motion during wide range of walking speeds (up to 3 MPH). The fluoroscopic system has a low radiation dosage, is non-invasive, and can be constructed using any pair of readily available fluoroscopes. Since this technique reproduced the knee positions during gait using 3D anatomic models of the knee, 6DOF tibiofemoral joint kinematics can be accurately obtained. This technique can be used as an alternative option for treadmill gait analysis in healthy, injured, and surgically treated knees.

## Competing interests

The authors declare that they have no competing interests.

## Authors' contributions

All authors were directly involved in the experiments, data analysis, interpretation of results and preparation of the manuscript. All authors have reviewed the text of the manuscript and agree with publication in the present form. GL carried out scanning, recruited subjects, performed data analysis, prepared manuscript, and revised manuscript. MK carried out scanning, subject recruitment, image processing, preparation of the manuscript and editing. AH assisted with scanning, subject recruitment, image processing and data analysis. FL supervised data analysis and interpretation, advised co-authors in preparation and revision of the manuscript. SKV designed experiment, supervised data analysis and manuscript preparation and revision. HER designed experiment, supervised data analysis and manuscript preparation and revision.

## References

[B1] Chao EY, Zweifach B (1986). Biomechanics of the human gait. Frontiers in Biomechanics.

[B2] Lafortune MA, Cavanagh PR, Sommer HJ, Kalenak A (1992). Three-dimensional kinematics of the human knee during walking. J Biomech.

[B3] Bach BR, Mikosz RP, Andriacchi TP (1988). The influence of changing femoral attachment positions on force displacement characteristics of the anterior cruciate ligament. Trans ORS.

[B4] Blankevoort L, Huiskes R, de Lange A (1988). The envelope of passive knee joint motion. J Biomech.

[B5] Karrholm J (1989). Roentgen stereophotogrammetry. Review of orthopedic applications. Acta Orthop Scand.

[B6] Selvik G (1989). Roentgen stereophotogrammetry. A method for the study of the kinematics of the skeletal system. Acta Orthop Scand Suppl.

[B7] van Dijk R, Huiskes R, Selvik G (1979). Roentgen stereophotogrammetric methods for the evaluation of the three dimensional kinematic behaviour and cruciate ligament length patterns of the human knee joint. J Biomech.

[B8] Andriacchi TP, Alexander EJ, Toney MK, Dyrby C, Sum J (1998). A point cluster method for in vivo motion analysis: applied to a study of knee kinematics. J Biomech Eng.

[B9] Li G, Suggs J, Hanson G, Durbhakula S, Johnson T, Freiberg A (2006). Three-dimensional tibiofemoral articular contact kinematics of a cruciate-retaining total knee arthroplasty. J Bone Joint Surg Am.

[B10] Banks SA, Hodge WA (1996). Accurate measurement of three-dimensional knee replacement kinematics using single-plane fluoroscopy. IEEE Trans Biomed Eng.

[B11] Stiehl JB, Komistek RD, Dennis DA, Paxson RD, Hoff WA (1995). Fluoroscopic analysis of kinematics after posterior-cruciate-retaining knee arthroplasty. J Bone Joint Surg Br.

[B12] Li G, Wuerz TH, DeFrate LE (2004). Feasibility of using orthogonal fluoroscopic images to measure in vivo joint kinematics. J Biomech Eng.

[B13] You BM, Siy P, Anderst W, Tashman S (2001). In vivo measurement of 3-D skeletal kinematics from sequences of biplane radiographs: application to knee kinematics. IEEE Trans Med Imaging.

[B14] Li G, Velde SK Van de, Bingham JT (2008). Validation of a non-invasive fluoroscopic imaging technique for the measurement of dynamic knee joint motion. J Biomech.

[B15] Varadarajan KM, Moynihan AL, D'Lima D, Colwell CW, Li G (2008). In vivo contact kinematics and contact forces of the knee after total knee arthroplasty during dynamic weight-bearing activities. J Biomech.

[B16] Defrate LE, Papannagari R, Gill TJ, Moses JM, Pathare NP, Li G (2006). The 6 degrees of freedom kinematics of the knee after anterior cruciate ligament deficiency: an in vivo imaging analysis. Am J Sports Med.

[B17] Zihlmann MS, Gerber H, Stacoff A, Burckhardt K, Szekely G, Stussi E (2006). Three-dimensional kinematics and kinetics of total knee arthroplasty during level walking using single plane video-fluoroscopy and force plates: a pilot study. Gait Posture.

[B18] Bohannon RW (1997). Comfortable and maximum walking speed of adults aged 20–79 years: reference values and determinants. Age Ageing.

[B19] Lelas JL, Merriman GJ, Riley PO, Kerrigan DC (2003). Predicting peak kinematic and kinetic parameters from gait speed. Gait Posture.

[B20] Miyoshi T, Shirota T, Yamamoto S, Nakazawa K, Akai M (2004). Effect of the walking speed to the lower limb joint angular displacements, joint moments and ground reaction forces during walking in water. Disabil Rehabil.

[B21] Andriacchi TP, Ogle JA, Galante JO (1977). Walking speed as a basis for normal and abnormal gait measurements. J Biomech.

